# *In Vitro* Antioxidant, Anti-inflammatory, and *In Vivo* Anticolitis Effects of Combretin A and Combretin B on Dextran Sodium Sulfate-Induced Ulcerative Colitis in Mice

**DOI:** 10.1155/2020/4253174

**Published:** 2020-11-07

**Authors:** Mbiantcha Marius, Dawe Amadou, Atsamo Albert Donatien, Ateufack Gilbert, Yousseu Nana William, Khalid Rauf, Mehreen Arif, Fanta Yadang Sabine Adeline, Njoku Isaac Saint, Hamza Dar, Naeem Ur Rehman, Izhar Ahmad

**Affiliations:** ^1^Laboratory of Animal Physiology and Phytopharmacology, Faculty of Science, University of Dschang, P.O. Box 67, Dschang, Cameroon; ^2^Department of Chemistry, Higher Teachers Training College, University of Maroua, P.O. Box 55, Maroua, Cameroon; ^3^Laboratory of Animal Physiology, Faculty of Science, University of Yaounde I, PO Box 812, Yaound'e, Cameroon; ^4^Department of Pharmacy, COMSATS University Islamabad, Abbottababd Campus, 22060 Abbottabad Khyber Pakhtunkhwa, Pakistan; ^5^Center for Research on Medicinal Plants and Traditional Medicine, Institute of Medical Research and Medicinal Plants Studies, P.O. Box 13033, Yaounde, Cameroon; ^6^Department of Chemistry, University of Lagos, Akoka, Lagos, Nigeria

## Abstract

*Combretum fragrans* (Combretaceae) is a Cameroonian medicinal plant containing various secondary metabolites and traditionally used for the treatment of several pathologies. Two cycloartane-type triterpenes, Combretin A and Combretin B, were isolated from this plant. This study was aimed at evaluating the anti-inflammatory, antioxidant, and anticolitis effects of these compounds. *In vitro* anti-inflammatory properties were evaluated by inhibition of cyclooxygenase, 5-lipoxygenase, and denaturation of the protein; antioxidant properties were assessed by using 1,1-diphenyl-2-picrylhydrazyl (DPPH), (2,2'-azino-bis(3-ethylbenzthiazoline-6-sulphonic acid)) ABTS^•+^, capacity tests ferric reducing antioxidant (FRAP), and trapping nitric oxide. For *in vivo* analysis, we used the model of ulcerative colitis induced by Dextran Sulfate Sodium (DSS). Studies of the anti-inflammatory activity showed that Combretin A and Combretin B had maximal inhibitory activity on cyclooxygenase (71.92% and 89.59%), 5-lipoxygenase (76.68% and 91.21%), and protein denaturation (63.93% and 87.78%). Antioxidant activity on DPPH, ABTS^•+^, ferric reducing antioxidant capacity (FRAP), and nitric oxide scavenging showed that Combretin A and Combretin B showed good antioxidant activities. These compounds significantly reduced the signs of DSS-induced colitis in the treated animals by preventing the weight loss of the animals, by significantly reducing the disease activity index, improving the condition of the stool, preventing the reduction of the length of the colon, and preventing the degradation of the colon. This study revealed that Combretin A and Combretin B have anti-inflammatory, antioxidant, and curative properties against colitis experimentally induced by DSS in rats.

## 1. Introduction

Inflammatory bowel diseases, mainly represented by ulcerative colitis and Crohn's disease, are chronic pathologies of the cell-mediated digestive tract which are accompanied by significant recurrent and chronic inflammation [1]. People with inflammatory bowel disease present a significant dysfunction of the immune system [2], and COX plays a very important role in the development and maintenance of ulcerative colitis [3]. In mammals, COX has two isoforms, COX-1 which is constitutive and COX-2 which is induced by the inflammatory response [3]. During the pathophysiology of inflammatory bowel diseases, inflammatory lesions trigger innate immune responses resulting in the production of chemokines and cytokines (TNF-*α*, IL-6, IL-1*β*) by intestinal epithelium cells and the macrophages that will be responsible for the development of the adaptive immune system [4]. Thus, in the absence of treatment, intestinal lesions can occur following an exaggeration of the inflammatory response [5]. Numerous studies have shown that several natural compounds which have the capacity to inhibit the production of cytokines and the activity of COX-2 have an important therapeutic efficacy against inflammatory bowel diseases [6].

When our body is attacked by bacteria and viruses, the inflammatory process is activated with the production of white blood cells and other protective substances [7]. These inflammatory reactions set up to protect our organism which involves reactive oxygen species starting with the activation of leukocytes. Thus, the effect of a product for the modulation of inflammatory processes could be provided by its antioxidant properties [8]. The release of elements such as hydroxyl radicals, superoxide, and/or peroxyl radicals is responsible for the development of oxidative stress. These reactive oxygen species play a very important role in the pathophysiology of many diseases (coronary artery disease, neurodegenerative disorders, aging, atherosclerosis, inflammation, chronic pain, Alzheimer's disease, cancer, and cataracts) [9, 10]. During the development of many inflammatory disorders, the phagocytes are activated; hydrogen peroxides (free radical species) such as the O_2_ and ∙OH radicals are released [11] and destroy the membrane by lipid peroxidation following a direct or indirect oxidative action. Tissue damage leads to the development of an inflammatory reaction with the production of chemotactic factors and numerous mediators [12].

In order to carry out scientific studies on inflammatory bowel disease, several animal models have been developed such as models transferred by adoption, chemically induced models, genetically modified models, and spontaneous models [13]. The DSS-induced colitis model (Dextran Sodium Sulfate), which is a very simple and reproducible model, is commonly used to assess the pathogenesis of ulcerative colitis and also to test new treatments potentially effective against ulcerative colitis [14]. Indeed, concerning this model, 1 to 5% of DSS is added to the drinking water which is given to the animals for several days; after a few days, animals develop symptoms such as diarrhea, loss of body weight, ulcers, bleeding, and mucosal lesions that are characteristics of human ulcerative colitis [15]. These symptoms can be relieved with many drug treatments; however, these different modern treatments have many side effects and increased tolerance in patients with ulcerative colitis [16]. For these different reasons, new therapeutic approaches remain to be developed and are currently necessary for the relief of people suffering from ulcerative colitis.

In the treatment of inflammatory disorders, a product capable of trapping reactive oxygen species, inhibiting the production of cytokines and COX, can be very beneficial. *Combretum fragrans* F. HOFFM (Combretaceae), a plant containing several classes of compounds such as saponins, steroids, flavonoids, triterpenes, and tannins [17, 18], is used in Africa to treat diarrhea, leprosy, pain, cough, inflammation, and chronic diabetic wounds. Two cycloartane-type triterpenes named Combretin A and Combretin B were isolated from the leaves of this species and showed *in vitro* inhibitory properties for the production of NO, TNF alpha, ROS, and immunomodulatory properties; they also showed analgesic, anti-inflammatory, antihypernociceptive, and neuroprotective properties [18–20]. However, the ability of Combretin A and Combretin B to inhibit the onset of symptoms of DSS-induced colitis has not been studied. The results of our previous work prompted us to check whether Combretin A and Combretin B could have anti-inflammatory activity in the model of DSS-induced ulcerative colitis in mice.

## 2. Methods

### 2.1. Reagents, Chemicals, and Equipment

1,1-Diphenyl-2-picrylhydrazyl (DPPH) was obtained from Sigma Aldrich (St. Louis, MO, USA). Sodium nitroprusside was purchased from Merck Ltd., India, Mumbai. Phosphoric acid, sodium hydroxide, sodium linoleate, sulfanilamide, sulfuric acid, linoleic acid, ascorbic acid, and arachidonic acid were purchased from SD Fine Chem. Ltd, Mumbai. Thiobarbituric acid, lipopolysaccharide, hemoglobin, ethylenediaminetetraacetic acid, and bovine albumin were obtained from Central Drug House Pvt. Ltd., New Delhi. Trypsin and casein were obtained from Hi-Media Lab. Ltd., Mumbai. *N*-(1-naphthyl)ethylenediamine dihydrochloride, potassium persulfate, potassium phosphate buffer, glutathione, and benzene were obtained from LOBA CHEME Pvt. Ltd. Mumbai. The chemicals include the following products: methanol, dimethyl sulfoxide (DMSO), and sodium chloride and were purchased from Geochim Sarl, west region of Cameroon. DSS was purchased from Tokyo Chemical Industry Co., Ltd., Japan. Prednisolone (Solupred) and all other chemicals and reagents were pure analytical grade and obtained from local suppliers.

### 2.2. Plant Material, Extraction, and Isolation

The plant material, referenced to the national herbarium of Cameroon with number 30309/H.N.C. in the name of *Combretum fragrans* (Combretaceae), was used to isolate the pure compounds tested in this study. The fresh leaves of this plant were harvested in the city of Maroua located in the far north region (Cameroon) [18]. These leaves were macerated in methanol; the extract obtained was successively fractionated in hexane, AcOEt, and BuOH. AcOEt fraction was subjected to conventional separation methods, and the isolated compounds (Combretin A and Combretin B) were described by usual spectroscopic methods [18].

### 2.3. *In Vitro* Assay

To develop the methodology of the study, Nguemnang et al. [21] was briefly followed.

#### 2.3.1. Anti-inflammatory Activity

*(1) Inhibition of Protein Denaturation*. For the evaluation of anti-inflammatory properties, the protocol described by Padmanabhan and Jangle [22] was used with some modifications. One milliliter of test compounds or diclofenac sodium in different concentrations (100, 200, 500, and 1000 *μ*g/mL) was added to 1 mL aqueous solution of 5% bovine serum that was then incubated for 15 minutes at 27°C. Distilled water and BSA mixture were used as control. For protein denaturation, the whole mixture was placed at 70°C for about 10 minutes. When the mixture is cooled down to room temperature, the activity of each mixture was measured at 660 nm. All tests were performed in triplicate. The following formula was used to study percent inhibition:

(1) $$\begin{array}{c} \%\text{Inhibition}=\frac{\text{Absorbance of control}–\text{Absorbance of sample}}{\text{Absorbance of control}}\times 100. \end{array}$$

*(2) Assay of Cyclooxygenase and 5-Lipoxygenase Inhibition*. **Lymphocyte culture preparation**. For the culturing of human peripheral lymphocytes, RPMI 1640 (HIMEDIA) that was supplemented with streptomycin, penicillin, and fetal calf serum was used. Phytohemagglutinin (HIMEDIA) used mediated the cell proliferation. The culture was filtered (cellulose acetate 0.2 *μ*m pore, Sartorios) and incubated for 72 hours after the addition of plasma (1 × 10^6^ cells/mL). This culture was then activated by adding lipopolysaccharide (1 *μ*L) and incubated for 24 hours. After activation, Combretin A, Combretin B, and diclofenac were added in different concentrations (100, 200, 500, and 1000 *μ*g/mL) and incubated for 24 hours. These are then centrifuged for 10 minutes at 6000 rpm for sedimentation. The supernatant was removed, and cell lysis buffer was added (50 *μ*L) and again centrifuged at 6000 rpm for 10 minutes [23].

**Assay of Cyclooxygenase**. Cyclooxygenase assay was performed using Tris-HCl buffer, enzyme, hemoglobin, and glutathione. To this mixture, arachidonic acid and TCA (10% in 1 N HCl, 0.2 mL) were added and incubated for 20 minutes at 37°C. Then, to the mixture, TBA (0.2 mL) was added and heated in boiling water for 20 minutes. This is then cooled and centrifuged for 3 minutes at 1000 rpm. The supernatant was used to measure COX activity at 632 nm [23].

**Assay of 5-lipoxygenase**. Lipoxygenase assay was performed by mixing equal quantities of linoleic acid (70 mg) and tween 20 in 4 mL of nonoxygenated water. To this mixture, sodium hydroxide (0.5 N) was added, and nonoxygenated water is added in a quantity sufficient to make a volume up to 25 mL. The resulting solution will be divided into 0.5 mL portions and thoroughly rinsed with nitrogen and then left for freezing. A quartz cuvette (25°C) with 1 cm optical pathway made it possible to carry out a reaction. The optic density (OD) was measured at 234 nm with a mixture containing sodium linoleate (0.2 mL), Tris buffer (2.75 mL, pH 7.4), and enzyme (50 mL) [23]. The percent inhibition was calculated by using the following equation:

(2) $$\begin{array}{c} \%\text{inhibition}=\frac{{\text{OD}}_{\text{control}}-{\text{OD}}_{\text{sample}}}{{\text{OD}}_{\text{control}}}\times 100. \end{array}$$

#### 2.3.2. Antioxidant Activity

*(1) DPPH Radical Scavenging Activity*. DPPH 24 mg was dissolved in 100 mL methanol; this solution is then diluted to a point to obtain absorbance at 517 nm of about 0.98 ± 0.02 forming a working solution. From this working solution, 3 mL was mixed with 100 *μ*L of Combretin A, Combretin B, or standard solution (1 mg/mL), and absorbance was measured (517 nm for 30 minutes) [24]. The control contained 100 *μ*L methanol in place of the plant sample. The percentage of antiradical activity (antioxidant) was determined by the following formula:

(3) $$\begin{array}{c} \%\text{antioxidant activity}=\frac{\text{Absorbance}\left(\text{control}\right)–\text{Absorbance}\left(\text{sample}\right)}{\text{Absorbance}\left(\text{control}\right)}\times 100. \end{array}$$

*(2) ABTS•+ Decolorization Assay*. ABTS (7 mM) in a volume of 9.5 mL was taken and mixed with 245 *μ*L of potassium persulfate (100 mM) that is supplemented to 10 mL with distilled water to form a working solution. This solution is left for 18 hours in dark at room temperature; after that, potassium phosphate buffer (0.1 M, pH 7.4) was introduced until an absorbance (734 nm) of about 0.70 ± 0.02 was obtained. The tested compounds were diluted in methanol to obtain concentrations of 0.1, 0.25, 0.5, and 1 mg/mL. 10 *μ*L of a sample was mixed with 2.99 mL of working solution and recorded at 734 nm [25]. The control was prepared by adding 10 *μ*L of methanol in place of the sample. The percentage of antioxidant activity was determined using the following formula:

(4) $$\begin{array}{c} \%\text{antioxidant activity}=\frac{\text{Absorbance}\left(\text{control}\right)–\text{Absorbance}\left(\text{sample}\right)}{\text{Absorbance}\left(\text{control}\right)}\times 100. \end{array}$$

*(3) Ferric Reducing Antioxidant Potential (FRAP)*. The working reagent was prepared by mixing acetate buffer (25 mL, 30 mM, pH 3.6), TPTZ (2.5 mL, 10 mM), and iron chloride (2.5 mL, mM) and incubated at 37°C (15 minutes). Concentrations between 50 mg/L and 500 mg/L of ascorbic acid produced a calibration curve [26]. The absorbance was measured at 593 nm for each specimen consisting of working reagent (2.85 mL), test compounds (150 *μ*L, 0.1 mg/mL) diluted in methanol, methanol (150 *μ*L), or stallion.

*(4) Nitric Oxide Scavenging Assay*. Sodium nitroprusside (0.5 mL, 5 mmol/L, pH 7.4) was combined at different concentrations 0.1, 0.25, 0.5, and 1 mg/mL of test compounds, ascorbic acid, or an equivalent amount of methanol and incubated for 180 minutes (25°C). Griess reagent (1.5 mL) consisting of sulfanilamide (1%), phosphoric acid (2%), and *N*-1-naphthylethylenediamine dihydrochloride (0.1%) was added, and the mixture was again incubated for 30 minutes. Absorbance was measured at 546 nm, and the percentage of activity was determined relative to the standard [27]. The following equation was used to show percent inhibition:

(5) $$\begin{array}{c} \%\text{nitric oxide scavenging}=\frac{\text{Absorbance of control}–\text{Absorbance of test}}{\text{Absorbance of control}}\times 100. \end{array}$$

### 2.4. *In Vivo* Assay

#### 2.4.1. Experimental Animals

The blind behavioral evaluation technique was performed for the administration of different treatments. Mice (males and females), aged about 3 months and weighing between 25 and 30 g, reared in an animal house (controlled temperature (22 ± 1°C) and a 12-hour light cycle/12 h in dark, with standard laboratory food and unlimited water) from the National Institutes of Health (NIH) Islamabad, Pakistan, were used for this test.

The minimum number of mice (6 animals per group) was granted by the National Institute of Health Institute (IACUC) Committee of National Institute of Health. In addition, the study protocols accepted by the Ethics Committee of the National Institute of Health Islamabad in Pakistan were used to assess the consistency of the effects of different treatments administered.

#### 2.4.2. Experimental Colitis

For the induction of experimental colitis, 4% DSS was used. Briefly, after an habituation period of one week, mice (42) were randomly divided into 7 groups (6 mice/group): untreated (no DSS), 4% DSS+2.5% DMSO/2.5% tween, 4% DSS+prednisolone (4 mg/kg), 4% DSS+Combretin A (25 mg/kg), 4% DSS+Combretin A (50 mg/kg), 4% DSS+Combretin B (25 mg/kg), and 4% DSS+Combretin B (50 mg/kg). The untreated group received distilled water without DSS. For other groups, water bottles were filled with 4% DSS. Combretin A, Combretin B, prednisolone, and distilled water were administered orally from the 5^th^ day after the onset of colitis induction by DSS.

Mice were scored daily for 10 days based on body weight and stool formation (bloody or nonbloody). Stool formation, bloody stool scores, and weight loss were averaged to determine the Disease Activity Index (DAI). Scores were assessed as follows: bloody stools (0: negative, 2: positive, or 4: gross bleeding), weight change (0: <1%, 1: 1–5%, 2: 5–10%, 3: 10–15%, or 4: >15%), and stool formation (0: normal, 2: loose stools, or 4: diarrhea). On day 10, the mice were sacrificed under ether vapor anesthesia and the intestines were isolated [28].

The entire colon from the ileocolic junction to the anus was quickly removed, opened longitudinally along the mesenteric fixation line, then washed with saline in a reservoir of ice water and dried with filter paper; then, the total length of the two points was measured [29].

#### 2.4.3. Macroscopic and Microscopic Evaluation of the Colon

The same colon removed, washed, and dried was photographed using a Canon camera (Cyber-shot, 7.2 megapixels, China); then, the samples from this colon were fixed in 10% formalin buffered with PBS. The sections were made in pieces of 5 *μ*m each; then, staining was done with hematoxylin-eosin (H&E) [29].

### 2.5. Statistical Analysis

All the data from *in vitro* tests indicate an average ± SEM of triplicate. The differences between groups were assessed by one way ANOVA followed by the Tukey posttest; for *in vivo* test, data are presented in the form of an average of 6 mice ± SEM, and the differences between groups were evaluated by ANOVA (two way) followed by Bonferroni posttest and ANOVA (one way) followed by Tukey's posttest. Significant differences were considered at *p* < 0.05.

## 3. Results

### 3.1. *In Vitro* Activity

#### 3.1.1. Anti-inflammatory Activity

*(1) Inhibition of Protein Denaturation*. Based on the results of this study, Combretin A and B effectively inhibited protein denaturation caused by heat.  Table 1 thus shows a significant (*p* < 0.001) inhibition of 63.93% and 87.78%, respectively, for Combretin A and B while diclofenac sodium at 1000 *μ*g/mL produced an inhibition of 89.20%.

*(2) Cyclooxygenase Inhibitory Assay*. Evaluation of cyclooxygenase activity determined the effect of both compounds on prostaglandin production. The results show that, at 1000 *μ*g/mL, Combretin A and B and Diclofenac sodium significantly inhibited (*p* < 0.001) the cyclooxygenase activity with 71.92%, 89.59%, and 97.88%, respectively (Table 1).

*(3) 5-Lipoxygenase Inhibitory Assay*. The evaluation of the activity of 5-lipoxygenase was used to study the effect of the compounds on the production of leukotrienes. It appears from this table that Combretin A and B, as well as diclofenac sodium, have a significant effect (*p* < 0.001) on 5-lipoxygenase activity with an inhibition of 76.68%, 91.21%, and 95.31%, respectively (Table 1).

#### 3.1.2. Anti-oxidant Activity

**Figure 1:**
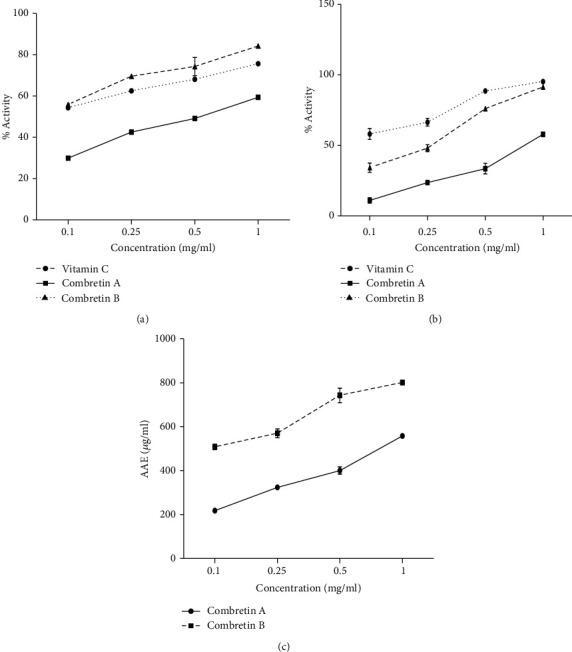
Antioxidant capacity of Combretin A and Combretin B on ABTS^•+^ radical assay (a) expressed as percentage activity, and on DPPH assay (b) as a function of time for 30 min and FRAP assay in terms of Ascorbic Acid Equivalents (AAE). Each point represents the mean of three replications.

*(1) ABTS•+ Radical Decolorization Assay*. It appears from Figure 1(a) that Combretin A and B and vitamin C (used as a reference substance) showed significant antioxidant activity on the relative recovery capacity of ABTS generated in the aqueous phase.

*(2) DPPH Radical Scavenging Activity*. The results presented in Figure 1(b) show the activity of Combretin A and B in free radical scavenging by the DPPH test. It appears that Combretin A and B and vitamin C at the concentration of 1 mg/mL showed a significant activity (*p* < 0.001) with inhibition percentages, respectively, of 91.38%, 57.83%, and 95.15%.

*(3) Ferric Reducing Antioxidant Potential (FRAP)*. The antioxidant capacity of Combretin A and B was determined using the FRAP assay (Figure 1(c)). Combretin B showed the highest and most significant antioxidant capacity (*p* < 0.001), followed by Combretin A. Both compounds exhibited a similar upward trend in activity with an increased concentration.

*(4) Nitric Oxide Scavenging Assay*.  Table 2 shows the comparison between the inhibition percentages of the compounds tested with ascorbic acid. This table shows that Combretin B activity was significantly greater than that of Combretin A in nitric oxide scavenging and that the activities of both compounds increased with increasing dose.

### 3.2. *In Vivo* Activity

#### 3.2.1. Effect of Combretin A and Combretin B on Body Weight

**Figure 2:**
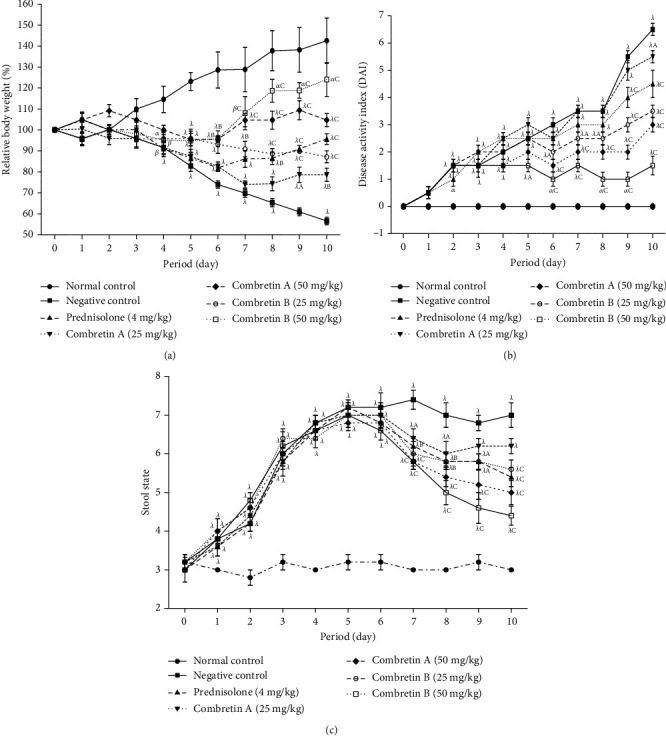
Effect of Combretin A and Combretin B on relative body weight (a), disease activity index (DAI) (b), and stool state (c) in DSS-induced colitis in rat. Values are expressed as mean ± SEM for six animals and analyses by two-way ANOVA followed by Bonferroni post hoc test. ^A^*p* < 0.05, ^B^*p* < 0.01, ^C^*p* < 0.001: significant difference compared with negative control;*^α^p* < 0.05, *^β^p* < 0.01, *^λ^p* < 0.001: significant difference compared with normal control.

In the group treated with DSS, the recorded weight loss was significant (*p* < 0.001) from day 4 after administration of DSS (4%), and this loss remained significant (*p* < 0.001) until day 10. Treatment with Combretin A and B (25 and 50 mg/kg) reversed weight loss from day 7. The effect of Combretin A and B (50 mg/kg) was greater than that of prednisolone (4 mg/kg), used as a reference substance (Figure 2(a)).

#### 3.2.2. Effect of Combretin A and Combretin B on the Disease Activity Index

The disease activity index scores increased significantly (*p* < 0.001) in all animals treated with DSS from the 2^nd^ day, as indicated by the incidence of diarrhea, weight loss, and bloody stools. However, Combretin A and B (50 mg/kg) and prednisolone (4 mg/kg) showed significant (*p* < 0.001) reduction of the severity of DSS-induced disease (Figure 2(b)).

#### 3.2.3. Effect of Combretin A and Combretin B on Stool Condition

Figure 2(c) shows the stool condition of animals treated with Combretin A and B (25 and 50 mg/kg) and prednisolone (4 mg/kg). It appears that the score increased significantly (*p* < 0.001) in all the animals treated on day 1 after the induction of colitis compared to the animals in the normal control group. On the 7^th^ day, the score of animals treated with the different compounds and with prednisolone was significant (*p* < 0.001) compared to that of animals in the negative control group. In addition, the effect of Combretin A and B (50 mg/kg) was greater than that of prednisolone.

#### 3.2.4. Effect of Combretin A and Combretin B on the Length of the Colon

**Figure 3:**
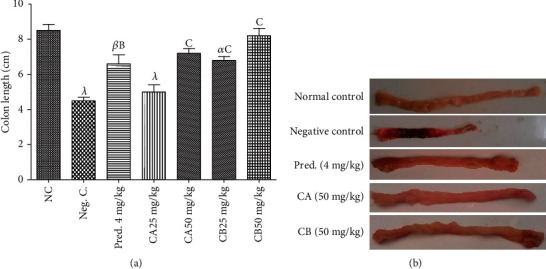
Effect of Combretin A and Combretin B on colon length in DSS-induced colitis in rats. Mice colons were isolated 10 days after DSS administration, and their lengths were measured. Values are expressed as mean ± SEM for six animals and analyses by one-way ANOVA followed by Tukey post hoc test; ^B^*p* < 0.01, ^C^*p* < 0.001: significant difference compared with negative control (Neg. C);*^α^p* < 0.05, *^β^p* < 0.01, *^λ^p* < 0.001: significant difference compared with normal control (NC). CA: Combretin A; CB: Combretin B.

Regarding the length of the colon, we found that the colon was significantly (*p* < 0.001) shorter in the negative control than in the normal control. Interestingly, treatment with Combretin A and B (50 mg/kg, *p* < 0.001) and prednisolone (4 mg/kg, *p* < 0.01) prevented the shortening of the colon significantly (Figure 3(a)).

#### 3.2.5. Macroscopic Assessment of Colon Damage

Figure 3(b) shows the macroscopic presentation of the intestine of the animals after the induction of colitis induced by DSS. DSS is found to cause bleeding development in animals in the negative control group. In contrast, the animals that received Combretin A and B (50 mg/kg) and prednisolone show no bleeding.

#### 3.2.6. Effect of Combretin A and Combretin B on the Histopathological Score

**Figure 4:**
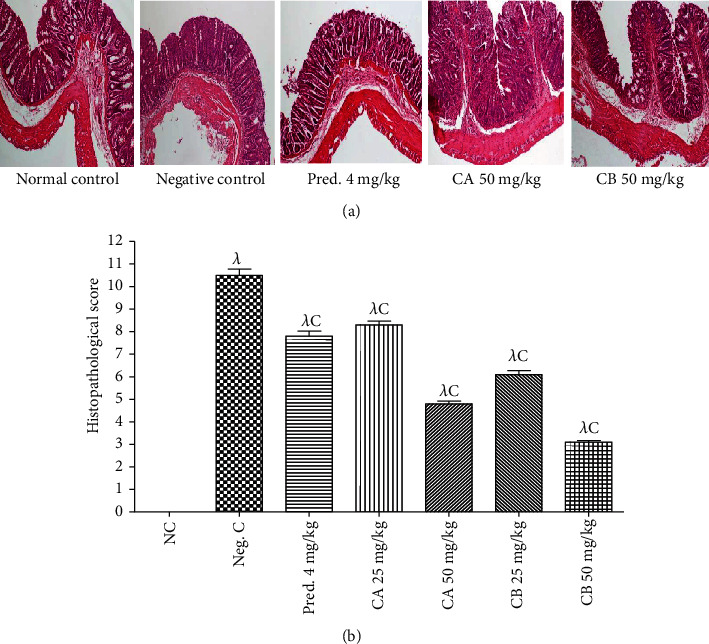
Effect of Combretin A and Combretin B on histopathological score in DSS-induced colitis in rat. (a) Representative hematoxylin and eosin (H&E)-stained distal colon sections and (b) the histologic scores of rats. Values are expressed as mean ± SEM for six animals and analyses by one-way ANOVA followed by the Tukey post hoc test; ^C^*p* < 0.001: significant difference compared with negative control (Neg. C); *^λ^p* < 0.001: significant difference compared with normal control (NC). CA: Combretin A; CB: Combretin B.

We next performed a histological analysis by performing H&E staining. As shown in Figures 4(a) and 4(b), 4% DSS treatment induced an inflammatory response that affected colonic architecture, cell infiltration, crypt shortening or loss, and goblet cell depletion. Administration of Combretin A (50 mg/kg), Combretin B (50 mg/kg), and prednisolone (4 mg/kg) significantly reduced the number of infiltrating cells, mucosal injury, and bleeding.

Histological examination of the colonic sections under light microscopy revealed that the untreated DSS mice exhibited typical inflammatory changes in the colonic architecture; the damage was assessed from histopathological scores for the colon (range 0 to 9). Compared with the normal control, the negative control mice showed a significant increase in the histopathological score of the disease (Figure 4). After treatment with Combretin A and B (25 and 50 mg/kg) and prednisolone (4 mg/kg), the histopathological score of the disease decreased considerably showing a protective effect of the compounds against damage caused by DSS.

## 4. Discussion

In the present study, the results indicate that Combretin A and B possess good anti-inflammatory properties; however, Combretin B showed the best anti-inflammatory activity on several *in vitro* models such as inhibition of protein denaturation, inhibition of 5-LOX, and COX inhibition. Several anti-inflammatory drugs such as diclofenac, phenylbutazone, salicylic acid, and many more have shown an important ability to dose-dependently inhibit heat-induced protein denaturation [30]. The denaturation mechanism is involved in the alteration of electrostatic force, hydrogen, hydrophobic, and disulfide bonds [31]. Steroidal and nonsteroidal anti-inflammatory drugs, commonly used for the management of inflammatory conditions, have the ability to bind to plasma albumin, preventing or inhibiting thermal denaturation of albumin [32]. Thus, in this study, diclofenac, used as a reference substance, significantly inhibited the denaturation of proteins. Known as a truly complex physiopathological response, inflammation involves a large production of free radicals derived from neutrophils, nitric oxide (NO), reactive oxygen species (ROS), prostaglandins, and cytokines during its process [33]. The pathogenesis of inflammatory diseases involves the overproduction of substances such as prostaglandin I2, prostaglandin E2, thromboxane A2, and leukotrienes from arachidonic acid through two metabolic pathways, the COX (cyclooxygenase) pathway and the 5-LOX (5-lipoxygenase) pathway [34]. Substances capable of inhibiting the activities of COX and 5-LOX resulting in a significant reduction in the production of leukotrienes and prostaglandins produce a large spectrum of anti-inflammatory activity and can be considered as having an excellent pharmacological safety profile in the clinic [35]. Ibuprofen, a nonsteroidal anti-inflammatory drug, is an inhibitor of COX which rapidly and reversibly and/or irreversibly seizes the active site of COX and acts as a competitive inhibitor of arachidonic acid oxygenation [36–38]. The inhibitory activity of Combretin A and B on protein denaturation, the cyclooxygenase pathway, and 5-lipoxygenase show that these two compounds are capable of significantly inhibiting the production of prostaglandins and leukotrienes, which confers to these two compounds anti-inflammatory properties. In addition, our previous work has shown that Combretin A and B are able to inhibit carrageenan-induced inflammation in rats *in vivo* and to inhibit the production of ROS, TNF-*α*, and NO *in vitro* [19].

Antioxidant substances as well as free radical scavengers are very often used to mitigate certain inflammatory pathologies; since, during the inflammatory process, there is a large production of free radicals that have the power to maintain the skin process [39]. In this study, several tests were used to evaluate the antioxidant activity of Combretin A and B; it was to put in the presence of compounds the reaction of the Fe^3+^/ferricyanide complex reduction in a ferrous form (ABTS test), the ion reduction reaction compounds with the formation of a ferric tripyridyltriazine complex (Fe^2+^-TPTZ) (FRAP test), and the radical reduction reaction DPPH [40, 41]. Combretin A and B showed a very important antioxidant capacity in all test models used with a concentration-dependent activity. During the development of the inflammatory process, macrophages are activated with overproduction of NO from nitric oxide synthase (iNOS) which will be responsible for an increase in vasodilation, vascular permeability, endothelial and tissue lesions, and development of inflammation. In addition to its important proinflammatory role in the development of chronic inflammation, NO is a diffusible free radical, considered a pleiotropic inhibitor in neuronal signaling, regulation of cell-mediated toxicity, relaxation of smooth muscle, platelet inhibition, and as an effector molecule in biological systems such as antimicrobial and antitumor activities, neuronal messages, or vasodilation [42]. From this study, Combretin A and B significantly reduced the amount of nitrite produced by the decomposition of sodium nitroprusside *in vitro*, suggesting that the direct NO scavenging process may be responsible for NO deletion by both compounds.

In the present study, the administration of 4% DSS in drinking water led to the development of symptoms of ulcerative colitis (bloody diarrhea, weight loss, and shortening of the length of the colon) in all the mice in the group untreated; moreover, mice having received DSS and untreated also presented a higher DAI score and a histopathological score compared to normal mice. In this study, we showed that Combretin A and B inhibited experimental colitis, resulting in an overall attenuation of DAI inflammation, including colon length and changes in body weight. Oral administration of 4% DSS in mice causes acute colitis. Animals have shown a variety of clinical symptoms of inflammatory bowel disease, including diarrhea, bloody stools, and weight loss. A disease activity index score was used as a reliable tool to assess the extent of gastrointestinal disease in experimental DSS-induced colitis. At the end of the experimental period, clinical symptoms were more pronounced. Prednisolone used in this study as the reference drug exerts its anti-inflammatory and immunosuppressive effect by inducing the gene expression of annexin therefore which have the main function the inhibition of the phospholipase A2 enzyme with the consequence of suppressing the synthesis of prostaglandins and leukotrienes by the arachidonic acid. Prednisolone can also act on macrophages and monocytes by blocking the synthesis and release of many proinflammatory cytokines (interleukin 1 (IL-1), interleukin 6 (IL-6), and tumor necrosis factor-*α* (TNF-*α*)) [43]. In the study, prednisolone significantly reduced all the symptoms of DSS-induced colitis. However, mice treated with Combretin A and B significantly improved the clinical score, suggesting a protective effect of these compounds in this model of colitis. Histological scores indicate that treatment with Combretin A and B reduced the damage to the mucous membranes. Ultimately, the results obtained indicate that treatment with Combretin A and B effectively attenuates established colonic inflammation and has a pronounced protective effect against colitis induced by DSS in mice.

Many cytokines such as TNF-*α*, IL-6, and IL-1*β* play a key role in the pathogenicity of inflammatory bowel disease, in regulating inflammation of the intestinal mucosa, and in the integrity of the intestinal epithelium [44, 45]. In addition, in people suffering from ulcerative colitis, the levels of TNF-*α*, IL-6, and IL-1*β* are greatly increased in the mucosa and serum [46]. Several studies have also shown that COX-2 expression is significantly elevated in animals with ulcerative colitis [47]. In addition, COX-2 and iNOS, known as inducible enzymes expressed rapidly by mononuclear macrophages, fibroblasts, or other cells after being stimulated in the body, are closely associated with the pathogenesis of ulcerative colitis. When iNOS and COX-2 are excessively released, oxidative damage can occur [48]. Likewise, ulcerative colitis is a peroxidative disease of the colon, and there are many intestinal pathological changes associated with the colitis process [49]. Oxidative stress is a response of the body stimulated by DSS. It can suppress endogenous defense systems that regulate the production of ROS [50]. High levels of ROS would have caused oxidative stress and DNA damage due to an imbalance between innate and exogenous antioxidants and ROS [51]. For these different reasons, several studies have shown that the regulation of these different pathways can be a solution for new therapeutic approaches against ulcerative colitis; thus, resveratrol exerts its effect against ulcerative colitis by increasing the expression of the SIRT1 gene, inducing the drop in the expression of p-IkB*α* as well as of COX-2 [52], while proto-catechic acid inhibits the production of inflammatory cytokines as well as COX-2 and iNOS to relieve patients with ulcerative colitis [53]. In previous studies, Combretin A and Combretin B have shown their ability to inhibit the production of NO, TNF alpha, and ROS, as well as their immunomodulatory properties [19], while in the present study, Combretin A and B have shown anti-inflammatory and antioxidant properties. These results suggest that the effect of Combretin A and Combretin B against DSS-induced ulcerative colitis may be closely associated with their regulatory effect on inflammatory cytokines, iNOS activity, COX-2 expression, ROS, and the antioxidant system.

## 5. Conclusion

The anti-inflammatory and antioxidant properties *in vitro*, followed by the *in vivo* anticolitic properties of Combretine A and B, were demonstrated in this study. The anti-inflammatory potentials of Combretin A and Combretin B were clearly described in the test for inhibition of denaturation of proteins, cyclooxygenase, and 5-lipoxygenase. The potential for trapping antioxidants and free radicals is clearly revealed in the DPPH, ABTS^•+^, power reduction, and nitric oxide trapping tests. We have shown that Combretin A and Combretin B exert an inhibitory effect on the pathogenesis of colitis, improving colon shortening, body weight, DAI, and histopathological scores in mice with DSS-induced colitis.

## Figures and Tables

**Table 1:** Effect of Combretin A and Combretin B on protein denaturation, cyclooxygenase (COX), and 5-lipoxygenase (LOX) inhibition.

Treatment	Dose (*μ*g/mL)	Activity			Inhibition (%)		
		Protein denaturation	COX	5-LOX	Protein denaturation	COX	5-LOX
Control	-	0.518 ± 0.004	-	-	-	-	-
Diclofenac	100	0.133 ± 0.002^c^	-	-	74.41	-	-
	200	0.121 ± 0.002^c^	-	-	76.72	-	-
	500	0.133 ± 0.002^c^	-	-	81.48	-	-
	1000	0.056 ± 0.001^c^	-	-	89.19	-	-
Ibuprofen	100	-	0.11 ± 0.006	0.232 ± 0.022	-	83.51	83.16
	200	-	0.018 ± 0.001^c^	0.038 ± 0.001^c^	-	88.79	87.41
	500	-	0.011 ± 0.44^c^	0.029 ± 0.005^c^	-	93.06	91.36
	1000	-	0.007 ± 0.001^c^	0.021 ± 0.006^c^	-	97.51	95.65
Combretin A	100	0.298 ± 0.011^c^	0.055 ± 0.009^b^	0.110 ± 0.009^b^	42.49	47.58	44.25
	200	0.255 ± 0.032^c^	0.041 ± 0.001^c^	0.075 ± 0.007^c^	50.92	59.58	60.87
	500	0.231 ± 0.023^c^	0.037 ± 0.003^c^	0.064 ± 0.003^c^	55.41	63.06	67.64
	1000	0.187 ± 0.007^c^	0.029 ± 0.001^c^	0.046 ± 0.003^c^	63.93	71.92	76.68
Combretin B	100	0.211 ± 0.005^c^	0.039 ± 0.001^c^	0.073 ± 0.006^c^	59.23	61.94	62.22
	200	0.158 ± 0.012^c^	0.030 ± 0.003^c^	0.058 ± 0.007^c^	69.44	71.04	69.77
	500	0.115 ± 0.003^c^	0.020 ± 0.001^c^	0.038 ± 0.009^c^	77.82	80.58	81.15
	1000	0.063 ± 0.005^c^	0.011 ± 0.002^c^	0.018 ± 0.005^c^	87.78	89.59	91.21

Each value represents the mean ± SEM;^b^*p* < 0.001, ^c^*p* < 0.001: significant difference compared to the control group. The percentage values were obtained using various concentrations of test compounds, and readings are presented as the mean of triplicates.

**Table 2:** Effect of Combretin A and Combretin B on nitric oxide scavenging activity.

	Concentration (mg/mL)	Absorbance (660 nm)	Inhibition (%)
Control	-	1.697 ± 0.102	-
Vitamin C	0.1	0.800 ± 0.058^c^	52.86 ± 1.90
	0.25	0.400 ± 0.029^c^	76.27 ± 2.22
	0.5	0.193 ± 0.006^c^	88.56 ± 0.47
	1	0.099 ± 0.009^c^	94.08 ± 0.80
Combretin A	0.1	1.136 ± 0.055^b^	32.28 ± 6.65
	0.25	0.873 ± 0.062^c^	48.41 ± 3.34
	0.5	0.780 ± 0.075^c^	54.22 ± 1.59
	1	0.608 ± 0.092^c^	64.54 ± 3.16
Combretin B	0.1	0.719 ± 0.071^c^	57.79 ± 2.18
	0.25	0.467 ± 0.034^c^	72.09 ± 3.45
	0.5	0.380 ± 0.015^c^	77.48 ± 1.38
	1	0.137 ± 0.032^c^	91.81 ± 2.16

The values were obtained using various concentrations of test compounds, and reading is presented as mean ± SEM of triplicates. ^b^*p* < 0.01 and ^c^*p* < 0.001 significantly different compared to control.

## Data Availability

The datasets used and/or analyzed during the current study are available from the corresponding author on reasonable request.

## Abbreviations

5-LOX: 5-Lipoxygenase
ANOVA: Analysis of variance
COX: Cyclooxygenase
DAI: Disease activity index
DMSO: Dimethyl sulfoxide
DPPH: 1,1-Diphenyl-2-picrylhydrazyl
DSS: Dextran sulfate sodium
ECACC: European collection of cell cultures
FRAP: Ferric reducing antioxidant capacity
H & E: Hematoxylin and eosin
IL: Interleukin
NIH: National Institutes of Health
NO: Nitrite oxide
OD: Optic density
ROS: Reactive oxygen species
TNF-*α*: Tumor necrosis factor alpha.
